# In Vitro Evaluation of Chito-Oligosaccharides on Disappearance Rate of Nutrients, Rumen Fermentation Parameters, and Micro-Flora of Beef Cattle

**DOI:** 10.3390/ani14111657

**Published:** 2024-05-31

**Authors:** Jianfu He, Jing Li, Qian Gao, Weijun Shen, Wenchang Liu, Min Xia, Haixiang Xiao, Dingfu Xiao

**Affiliations:** 1 College of Animal Science and Technology, Hunan Agricultural University, Changsha 410128, China; hejianfu807@sina.com (J.H.); jing1173442331@outlook.com (J.L.); gaoqian996@sina.com (Q.G.); shenweijun@hunau.edu.cn (W.S.); bgmusic79@163.com (W.L.); xmin1017001@163.com (M.X.); xhx248260@sina.com (H.X.); 2 Yuelushan Laboratory, Changsha 410128, China

**Keywords:** beef cattle, chitosan oligosaccharides, RUSITEC system, rumen fermentation, microorganisms

## Abstract

**Simple Summary:**

This study investigated whether COS in the diet can improve the digestion and rumen health of beef cattle under in vitro fermentation conditions. A total of 24 fermentation tanks were randomly allocated into four groups. Each treatment had six replicates, with one tank per replicate. Each group received a different concentration of 0.02% COS, 0.04% COS, 0.08% COS, and a control group (CON). The results demonstrated that the addition of COS facilitates a shift in rumen fermentation from the acetate model to the propionate model. This is achieved by influencing the microbial population, resulting in alterations to the fermentation level, nutrient disappearance rate, and gas production. These changes have the potential to enhance rumen health.

**Abstract:**

The study aimed to investigate the effect of dietary chitosan oligosaccharides (COS) meal levels on the nutrient disappearance rate, rumen fermentation, and microflora of beef cattle in vitro. A total of 24 fermentation tanks were randomly divided into four treatments containing 0% COS (CON), 0.02% COS, 0.04% COS, and 0.08% COS for an 8-day experiment period, with each treatment comprising six replicates. The disappear rates of DM, CP, EE, and total gas production were quadratically increased with increasing COS levels. The disappear rates of DM, CP, EE, and ADF were greatest, whereas the total gas production was lowest in the 0.08% COS group. The pH, NH_3_-N, MCP, the content of propionate, isobutyrate, butyrate, valerate, and the A/P were quadratically increased with increasing COS levels, while the A/P were linearly decreased. The pH, MCP, and the content of propionate, and butyrate were highest, whereas the NH_3_-N and the content of acetate, isobutyrate, valerate, and the A/P were lowest in the 0.08% COS group. Microbiomics analysis showed that the rumen microbial diversity was not altered between the CON and the 0.08% COS group. However, the relative abundance of *Methanosphaera*, *Ruminococcus*, *Endomicrobium*, and *Eubacterium* groups was increased, and the relative abundance of pathogenic bacteria *Dorea* and *Escherichia-Shigella* showed a decrease in the 0.08% COS group. Overall, the 0.08% COS was the most effective among the three addition levels, resulting in an increase in the disappearance rate of in vitro fermented nutrients and improvements in rumen fermentation indexes and microbial communities. This, in turn, led to the maintenance of rumen health.

## 1. Introduction

A number of challenges have constrained the rapid development of beef cattle farming, including long-distance transport, stress caused by climate change, high feed prices, and low nutrient utilisation [1]. These factors have resulted in suboptimal growth performance, meat quality, and reduced economic benefits. In the previous studies, the rumen fermentation indicators and microbial community distribution are important indices to understand the health status of the rumen [2,3,4]. However, due to the special multi-stomach structure of ruminants, the rumen health of ruminants has always been a major concern in ruminant breeding [5]. However, the rumen has complex physiological functions and digestive characteristics [6], and the mechanism of these physiological functions has not been completely clarified so far.

In previous studies, people have tried almost all feed substitutes and achieved remarkable results, as well as tried different types of additives, which have been proven to improve animal growth performance improving growth and enhancing immunity [7,8,9,10]. Chitin is a natural polymer with high production and biodegradability in nature, second only to cellulose. It can be deacetylated to obtain chitosan [11], and COS can be prepared by enzymatic or chemical hydrolysis of chitosan [12]. The COS was an additive with multiple biological activities and functions, which was prepared by enzymatic or chemical hydrolysis of chitosan. Compared to chitosan, COS exhibits low molecular weight, high degree of deacetylation, high polymerisation, and low viscosity, and the molecular weight of COS is in the range of 0.5–2.5 kDA [13,14]. Therefore, COS not only has the biological effects of chitosan, but its effect is even stronger than that of chitosan [13]. The physicochemical properties of COS give it significant biological properties, including anti-oxidant, anti-inflammatory, drug, and *DeoxyriboNucleic Acid* (DNA) delivery capabilities [15]. Consistently, research has shown that COS can enhance animal growth performance, facilitate the development of immune organs, reinforce the small intestinal mucosal barrier function [16], and mitigate the inflammatory response of the intestinal tract [17], regulating gastrointestinal function [18]. However, the effect of COS on the rumen fermentation of beef cattle is not yet clear.

The rumen simulation technology (RUSITEC) system is an in vitro fermentation device that mimics the physiological functions of the rumen with the aim of reducing the limitations of in vivo experiments, such as inconsistencies in the genetic background and physiological state of individual animals [19]. The RUSITEC system plays an important role in the study of rumen microorganisms and the mechanism of rumen fermentation [20]. Therefore, in this study, the RUSITEC system was used to investigate the effects of different levels of COS added to diets on rumen fermentation of beef cattle and to evaluate whether COS can be used as a safe and effective feed additive, with particular attention on nutrient disappearance rates, rumen fermentation parameters and rumen microbial community.

## 2. Materials and Methods

### 2.1. Experimental Animals, Feeding Management, and Experimental Design

Three *Xiangxi* yellow cattle (*Bos taurus*; a native breed in Hunan Province, China) possessing rumen fistulas were enlisted as donors of rumen fluid, the weight of the experimental animals is 385 ± 28.7 (mean ± standard deviation) kg, with an age of 3 ± 0.5 years. Rumen fluid collection was followed by its deployment in the RUSITEC system for in vitro fermentation assays. Formulation of the diet and feeding amount for rumen fluid donors adhered to the nutritional benchmarks delineated in the Chinese Beef Cattle Feeding Standards (NY/T815-2004) [21]. The dietary regimen for the cattle was comprised of a blend of wheat straw and concentrates in a 1:1 ratio (Table 1). Feeding sessions were conducted at 08:00 and 18:00, restricted dietary feeding. The study utilised a single-factor randomised trial design, dividing 24 fermentation tanks into four groups, each group with six replicates. The substrate was the basal diets with supplemented 0.02%, 0.04%, and 0.08% COS, respectively, COS are provided by Zhongke Rongxin Biotechnology Co., Ltd. (Suzhou, China) with a purity of ≥85%, mixed well with the fermentation substrate by mixing step by step. The adaptation period lasted for five days and was then followed by a three-day sampling period. The experiment was conducted at the Animal Science and Technology Experimental Building and Animal Training Center of Hunan Agricultural University.

### 2.2. Rumen Simulation Technique Fermentation

The construction and operation programme of the RUSITEC system utilised in this experiment is detailed in the study by Adebayo [22]. In brief, the RUSITEC system is a dual-flow continuous culture system that simulates rumen fermentation in vitro. It primarily comprises a simulated fermentation device, a constant temperature device, an information processing section, and a fermentation parameter control section. The simulated fermentation device comprises a fermentation tank, stirrer, buffer container, water-cooled overflow bottle, gas collection bag, and gas, pressure, temperature, and pH sensors, which are primarily employed for the collection of pertinent parameters. The constant temperature device encompasses a constant temperature heating device and a reflux pipe, which are utilised to ensure that the fermentation process maintains a constant temperature. The information processing section comprises four modules: gas, pressure, temperature, and pH, which are utilised for the detection of relevant parameters. The fermentation parameter control section comprises an intelligent operating system, hardware motherboard, and chips, which are employed for the overall control and operation of the device. The fermentation temperature of the RUSITEC system’s fermentation tank can be maintained at 39 ± 0.5 °C. The McDougall [23] buffer solution is continuously injected into the fermentation tank at a set rate through a peristaltic pump, and the overflow liquid and undegraded solid components of each fermentation tank are automatically and continuously discharged into a water-cooled overflow bottle in a 4 °C water bath. The water-cooled overflow bottle is maintained at a low temperature to prevent further fermentation, thereby achieving a more accurate simulation of rumen fermentation in living animals.

Prior to the formal test, it is necessary to ascertain the airtightness of the RUSITEC system and conduct a trial run. During the formal experiment, the thermos was preheated to 39 °C and filled with CO_2_ in advance. Prior to the morning feeding, rumen contents were collected from three *Xiangxi* yellow cattle in a rumen fistula tube, filtered through four layers of sterilised gauze, and returned to the laboratory. A constant N_2_ airflow was then introduced into the device to maintain anaerobic conditions, while 500 mL of preheated filtered rumen fluid and 500 mL of McDougall buffer were introduced into each fermentation tank. Once the device has commenced operation, a constant N_2_ airflow should be introduced, after which 20 g of fermentation substrate (DM basis) should be added to the fermentation tank. This should be performed at 08:00 and 18:00 each day. The composition of the fermentation substrate should be consistent with that of the rumen fluid donor cow feed. Wheat straw and concentrate supplement are dried to a constant weight in a 65 °C constant temperature drying oven and then ground by a grinder through a 1 mm aperture sieve. They are mixed in a 1:1 ratio. The constant temperature device maintains a temperature of 39 ± 0.5 °C in the fermentation tank. The intelligent operating system controls the stepper motor to stir the contents of the fermentation tank at a rate of 25 r/min. The McDougall buffer is introduced into the fermentation tank via a pressure pump at a rate of 6% per hour. The overflow bottle conduit is monitored to ensure that the overflow liquid and undegraded solid phase fermentation substrate in each fermentation tank can be collected in a timely manner and terminated in the overflow bottle.

### 2.3. Sample Collection

Prior to the addition of substrate each morning, a 5 mL sample of fermentation broth was collected from the liquid phase sample collection port of the fermenter by filtering the collection through four layers of sterile gauze, which was then placed in a 50 mL centrifuge tube and added to 15 mL of methyl green staining solution. The methyl green staining solution was prepared by completely dissolving 6 g of methyl green and 8 g of sodium chloride in 1 L of 35% formaldehyde solution. The solution is then mixed well and placed in a dark location to shake and stored for protozoa counting. During the sampling period (days 6 to 8 of the experiment), the collected material in the water-cooled overflow bottle is filtered through a nylon bag before the addition of substrates. A solid phase sample is then collected. After cleaning, drying, crushing, and storage in a self-sealing bag, the sample is used to determine nutrient content and calculate the nutrient disappearance rate. Prior to the addition of substrates each morning during the sampling period, 15 mL of fermentation broth is collected from the liquid phase sample collection port of the fermentation tanks. The pH is then measured using a portable pH meter (PHS-3C, Shanghai Yidian Scientific Instrument Co., Ltd., Shanghai, China), after which 1.5 mL of the collected fermentation broth is divided into sterile and enzyme-free 2 mL centrifuge tubes. A total of 300 µL of 25% metaphosphate is added to the samples of two centrifuge tubes, which are then mixed and acidified uniformly. These samples are subsequently used for the determination of volatile fatty acids (VFA) and NH_3_-N concentrations, respectively. Prior to measurement, the samples were stored at a temperature of −20 °C. Two tubes of samples were used for microbial crude protein (MCP) determination and microbial omics analysis and were stored at −20 °C prior to measurement. The remaining samples were stored in a −80 °C refrigerator for future use. On the morning of the sampling period, after adding substrates, a 3 L gas bag was installed through the gas phase sample collection port to collect the discharged gas. The volume of gas collected in the bag is measured using a graduated syringe, and the total volume of gas collected is recorded.

### 2.4. Chemical and Biological Analysis

The determination of DM, ash, EE, and CP content in both substrate and fermentation residue followed the methods outlined by AOAC [24]. Moreover, the calcium (Ca) and phosphorus (P) content in the feed were assessed using the AOAC [24] method. The calculation method for organic matter (OM) is: $OM\left(\%\right)=1-ash\left(\%\right)$. The NDF and ADF content in both feed and fermentation residue were determined as described by Van Soest [25]. And the calculation method for the disappearance rate of nutrients is:

x=[(a×b−c×d)/(a×b)]×100%

where x: disappearance rate of nutrients; a: mass of substrate added every day, dry matter basis; b: measured value of a certain nutrient component in the substrate; c: mass of residue collected every day during the sampling period, dry matter basis; d: measured value of a certain nutrient component in the residue).

Protozoa enumeration was conducted using the Sedgewick Rafter counting plate. A proportional mixture of fermentation broth and methyl green staining solution was prepared, with 1 mL of the resultant solution transferred onto the counting plate. The plate was examined under an optical microscope according to Kisidayov’s methodology [26]. VFA concentration in the fermentation broth was determined via gas chromatography [27], NH_3_-N content was quantified using the phenol hypochlorite method [28], and MCP content was assessed following the method outlined by Makkar [29].

### 2.5. Microbiological Analysis of Fermentation Broth

Microbiological analysis of fermentation broth samples was conducted by Beijing Ovison Gene Technology Co., Ltd. (Beijing, China). The detailed purification, quantification, sequencing steps and analytical methods are consistent with those in our previous studies [30], as described in detail below.

Microbial genomic DNA was extracted from the fermentation liquid samples using the E.Z.N.A. Soil DNA Kit (Omega Bio-tek, Inc., Norcross, GA, USA), following the manufacturer’s instructions. The purity and concentration of the extracted DNA were determined using the TBS-380 and NanoDrop2000 spectrophotometers (Thermo Fisher Scientific, Waltham, MA, USA), respectively. Furthermore, the integrity of the extracted DNA was verified through 1% agarose gel electrophoresis. The DNA samples were subsequently stored at −80°C for subsequent experiments.

The rumen microbial community structure can be obtained by sequencing the region of the bacterial 16S rRNA gene and the eukaryotic 18S rRNA gene. The V3 and V4 hypervariable regions of the bacterial 16S rRNA gene were amplified using universal primers: 338F (5′-ACTCCTACGGGAGGCAGCAG-3′) and 806R (5′-GGACTACNNGGGTATCTAAT-3′). The V4 hypervariable region of the eukaryotic 18S rRNA gene was amplified using the primers 573F (5′-CGCGGTAATTCCAGCTCCA-3′) and 951R (5′-TTGGYRAATGCTTTCGC-3′). The purification, quantification, and sequencing of the 16S rRNA gene and 18S rRNA gene were conducted on the Illumina MiSeq/NovaSeq (Illumina, Inc., Albany, NY, USA) platform at Beijing Allwegene Technology Co., Ltd. (Beijing, China).

Based on previous studies [31,32], the original sequencing reads of the 16S rRNA gene and the 18S rRNA gene were demultiplexed, quality filtered, and merged. The qualified sequences were clustered into operational taxonomic units (OTUs) with 97% similarity using the Uparse algorithm of the Vsearch (v2.7.1) software and all OTU representative sequences were classified into different taxonomic groups against the Silva database using the BLAST tool. The *α*-diversity indices (Chao1, Observed_species, PD_whole_tree, and Shannon) were calculated based on the OTU information with the QIIME software (v1.8.0). The difference test of these indices between the two groups was conducted by the Wilcoxon rank sum test. A principal coordinates analysis (PCoA) was conducted based on the Bray–Curtis distance at the OTU level in order to assess β-diversity. Furthermore, an Adonis (PERMANOVA) analysis was conducted to assess significant differences in *β*-diversity of bacteria and protozoa between the two groups. The taxonomic annotation and relative abundance of microbial species at the phylum and genus levels were visualised as bar-plot diagrams using the R software (v3.6.0). The Linear Discriminant Analysis Effect Size (LEfSe) analysis was conducted using the Python (v2.7) software to identify the signature microbiota between the two groups.

### 2.6. Statistical Analysis of Data

**Figure 1:**
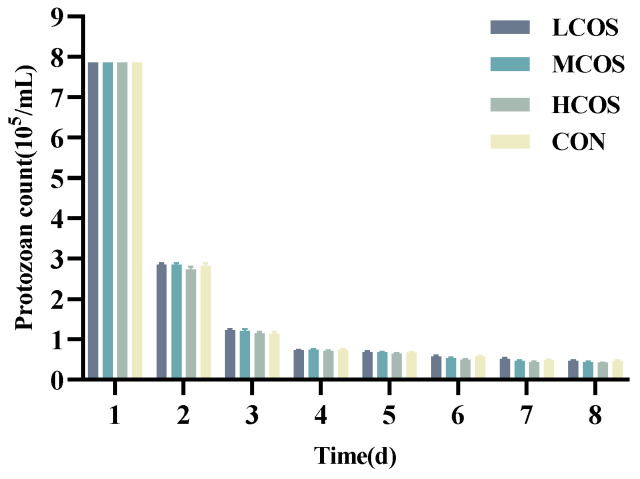
Dynamic changes in the number of rumen protozoa in four treatment groups on days 1–8.

**Figure 2:**
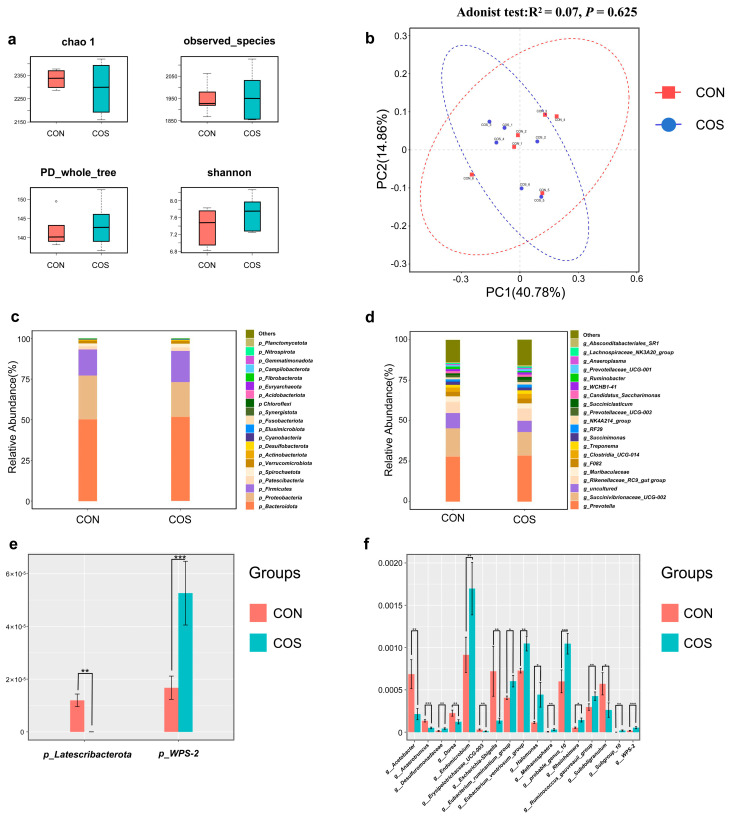
Effects of adding 0.08%COS to the diet on the rumen microbial community of beef cattle (*n* = 6). (**a**) α-Diversity index, including Chao 1, observed_species, PD_whole_tree, and Shannon. (**b**) *β*-Diversity index, PCoA analysis based on Bray-Curtis distance. The composition of rumen bacterial communities (top 20) at the phylum level (**c**) and genus level (**d**). The differential microbial communities between the two treatment groups at the phylum level (**e**) and genus level (**f**). And the “*” in (**e**,**f**), respectively, represent: * (0.05 < *p* < 0.1), ** (0.01 < *p* < 0.05), *** (*p* < 0.01).

Data analysis was performed using SPSS 20.0 software (SPSS, Inc., Chicago, IL, USA), which was employed for analysing data nutrient disappearance rate, VFA, NH_3_-N, MCP, gas production, and pH using single-factor random design. Results are presented as mean ± Standard Error of Mean. The Duncan multiple range test was utilised to determine the significance of differences between treatment groups. A *p*-value of less than 0.05 indicates significant difference, and less than 0.01 indicates extremely significant differences, while *p*-value between 0.05 and 0.1 indicates a trend. Additionally, GraphPad Prism 8.0 software (Origin, CA, USA) was utilised for data visualisation and plotting. Figure 1 was drawn using GraphPad Prism (9.5.0.730), all images (Figure 1 and Figure 2) are grouped and spliced using the PDF editing function in WPS Office (12.1.0.16729) and enhanced in pixel and resolution using Adobe Illustrator 2023.

## 3. Results

### 3.1. Rumen Protozoa Count

The number of rumen protozoa (Figure 1) in the four groups showed a rapid decline on day 2 after inoculation into the fermenters, followed by a slower decline on days 2–4 and a gradual stabilisation of the numbers after day 5. As shown in Table 2, during the initial five days of the experiment, when compared with the CON group, the inclusion of COS in the diet had no significant effect on the rumen protozoa count (*p* > 0.05). However, the 0.08% COS group witnessed a significant decline in protozoa count on the 6th and 8th day (*p* < 0.05), and there is a declining trend on the 7th day.

### 3.2. Nutrient Disappearance Rates and Total Gas Production

As shown in Table 3, the disappear rates of DM, OM, CP, and EE, and the total gas production of day 6, day 7, and day 8 were quadratically decreased (*p* < 0.05) with an increasing COS level. With the increased level of COS, the total gas production of day 6, day 7, and day 8 also linearly decreased (*p* < 0.05). The disappearance rate of DM, OM, CP, EE, and ADF was greatest (*p* < 0.05), whereas the total gas production was least (*p* < 0.05) in the 0.08% COS group. There were no differences (*p* > 0.05) in the disappearance rate of OM and NDF among the COS groups.

### 3.3. The rumen Fermentation Parameters

As shown in Table 4, the pH, NH_3_-N, MCP, the content of propionate, isobutyrate, butyrate, valerate, and the acetate/propionate (A/P) were quadratically increased (*p* < 0.05) with an increasing COS level. In addition, the A/P were linearly decreased (*p* < 0.05) with an increasing COS level. The pH, MCP and the content of propionate and butyrate were highest, while the NH_3_-N and the content of acetate, isobutyrate, valerate, and the A/P were lowest (*p* < 0.05) in the 0.08% COS group. There were no differences (*p* > 0.05) in the total VFA and the content of isovalerate.

### 3.4. The Rumen Microbial Community

Following the assessment of nutrient disappearance rates and rumen fermentation indicators, the addition level of 0.08% COS emerged as the most effective among the three treatment groups. Consequently, samples from the 0.08% COS and CON groups underwent analysis to examine the impact of COS on the rumen microbial community of beef cattle. There were no significant differences between the two groups in the α-diversity indices of Chao1, observed_species, PD_whole_tree, and Shannon (*p* > 0.05; Figure 2a). Additionally, the principal coordinate analysis (PCoA) based on the Bray–Curtis distance metric (Figure 2b) revealed that the addition of COS did not induce significant alterations in the composition of rumen bacteria (*p* > 0.05). At the phylum level, the top 20 rumen bacterial communities were evaluated in Figure 2c; predominant communities in both treatment groups included *Bacteroidota*, *Proteobacteria*, and *Firmicutes*. Two differential communities were identified, the relative abundance of the *Latescibacterota* in the COS group was significantly decreased compared to that in the CON group, whereas the relative abundance of the *WPS-2* was significantly increased in the COS group (*p* < 0.05; Figure 2e). Moreover, at the genus level, the top 20 rumen bacterial communities were evaluated in Figure 2d; predominant communities in both treatment groups included *Prevotella*, *Succinivibrionaceae_UCG-002*, and *Rikenellaceae-RC9_gut group*. Seventeen differential communities were discerned. Compared to the CON group, the relative abundance of *Desulfuromonadaceae*, *Endomicrobium*, *Eubacterium-ruminantium_group*, *Eubacterium-Venturiosum_group*, *Halomonas*, *Metanosphaera*, *Probable_genus_10*, *Rheinheimera*, *Ruminococcus_govreauii_group*, *Subgroup_10*, and *WPS-2* exhibited a significant increase (*p* < 0.05), and the relative abundance of *Acetobacter*, *Anaerotruncus*, *Dorea*, *Erysipelotrichaceae_UCG-003*, *Escherichia-Shigella*, and *Subdoligranulum* was decreased (*p* < 0.05) in the COS group (Figure 2f).

## 4. Discussion

Multiple studies have confirmed through in vitro and in vivo experiments that chitosan can be a safe and efficient feed additive [33,34,35,36]. The COS in this study is an oligomeric derivative of chitosan, obtained by deacetylation of chitin. It has the same safety profile as chitosan and a stronger biological activity and action than chitosan [37]. However, COS is rarely employed in beef cattle, and there has been no attempt to ascertain whether it can retain its biological activity in the complex digestive system of beef cattle. Therefore, the objective of this study was to determine whether COS can influence the rate of nutrient disappearance and the rumen fermentation process through an in vitro fermentation test.

### 4.1. Effects of Adding Different Levels of COS to the Diet on Rumen Protozoa Count of Beef Cattle In Vitro

The rumen is an important digestive organ in ruminants, and its stable internal environment, such as pH, microbial community, and VFA content, is a key factor in measuring the growth and health of ruminants [38]. Rumen protozoa are the largest microbial community in the rumen environment, and their quantity and morphology can be observed under a microscope through certain methods. It is essential that the RUSITEC system, which simulates the rumen digestion process, maintains high stability during operation in order to ensure the accuracy of experimental data. Therefore, it is possible to visually evaluate the operation of the system by collecting daily rumen fermentation broth and observing the number of protozoa in it. In this experiment, the number of protozoa in the fermentation broth exhibited a rapid decline between days 1 and 5, followed by a gradual stabilisation from day 6 to day 8 (Figure 1), which is consistent with the description provided by Li [39]. This suggests that samples can be collected when the number of protozoa is stable. As for the reason for the rapid decrease in the number of protozoa between day 1 and day 5, Shen [40] pointed out in his research that due to the need to add substrates twice a day in the continuous in vitro method, although N_2_ is introduced, it still cannot avoid contact with the air. The protozoa need an adaptive process, which leads to a rapid decrease in the number. And ultimately, this is an in vitro method, with an experimental period of up to eight days. Only the addition of rumen fluid on the first day, diluted with artificial saliva, can lead to a decrease in the number of protozoa. After the fifth day, the number of protozoa in this device has stabilised, indicating that it has adapted to the new environment. In this study, it was observed that the number of rumen protozoa in the 0.08% COS group was lower than that in the CON group during the sampling period. In previous studies on chitosan, similar results were obtained, namely that chitosan reduced the number of protozoa [33,41]. Previous studies have shown that changes in the number of rumen protozoa are related to eating and drinking behaviour [42,43], protein supply in the diet [44], and abundance of rumen bacterial communities [45]. Interestingly, although studies have shown a close correlation between the number of rumen protozoa and the proportion of nutrients in the rumen, the digestibility of nutrients, and the rumen microbiota [46,47], in this study, the adaptive period COS did not have a significant impact on rumen protozoa. However, during the sampling period, it was found that the number of protozoa with 0.08% COS was significantly lower or had a tendency to be lower than the CON group. It is possible that the addition of COS resulted in the consumption of nutrients in the fermentation substrate being accelerated in the early stages. When the number of rumen protozoa stabilises from the sixth day, the consumption of nutrients required for their growth in the pre-fermentation stage leads to a decrease in the number of protozoa.

### 4.2. Effects of Adding Different Levels of COS to the Diet on Nutrient Disappearance Rates and Total Gas Production of Rumen Nutrients in Beef Cattle In Vitro

In this study, it was observed that the disappearance rates of DM, OM, CP, EE, and ADF in the solid-state components of the fermentation broth increased to varying degrees after the addition of different proportions of COS, the 0.08% COS exhibited the best effects. In previous studies, Liu [48] observed that the addition of COS did not affect the apparent digestibility of the nutrients. Different results were also obtained in studies related to chitosan. Araújo [49] and Mingoti [50] observed that chitosan had no significant effect on the apparent digestibility of nutrients in beef cattle and cows. In contrast, Wenchelova [51] demonstrated that chitosan could result in a reduction in the apparent digestibility of nutrients in the rumen of sheep. Goiri [52] and Zhang [53] found that chitosan was able to improve rumen digestibility of nutrients in sheep. The different results may be due to the different diets in the tests, the different test animals, and the differences between in vivo and in vitro methods. Furthermore, the microbiological outcomes of this study indicate that COS had the potential to elevate the relative abundance of *Metanosphaera* and *Ruminococcus*. The *Metanosphaera* can promote the utilisation of fermentation substrates in the rumen [54], while the *Ruminococcus* can accelerate cellulose degradation, improve rumen digestion ability, and regulate rumen function [55,56]. In summary, the increased disappearance rate of rumen nutrients such as DM, CP, EE, and ADF can be explained by the increase in relative abundance of *Metanosphaera* and *Ruminococcus* caused by the addition of COS, which promotes the utilisation of nutrients such as DM, CP, EE, and ADF in the rumen. DM is closely related to OM, leading to an increase in the disappearance rate of OM. The increase in nutrient disappearance rate can promote the digestion of feed, improve feed utilisation efficiency, and promote animal growth.

The total gas production can be used to evaluate the efficacy of rumen fermentation and it is also one of the important indicators of equipment stability. Previous studies have indicated that total gas production is related to the degree of degradation of fermentation substrates [57]. In this study, the total gas production decreased with the increase in the addition of COS in the diet. In previous studies, chitosan led to a decrease [58,59] or no change [60] in CH_4_ production, and there seems to be no consistent conclusion. Liu [61] proposed that the change in acetate concentration is positively correlated with the abundance of acetate-type *Methanogens*. However, in this study, the concentration of acetate significantly decreased, and the total gas production also decreased. However, no significant change in the abundance of acetate-type methanogenic bacteria was observed in the results of microbiology. On the contrary, we observed an increase in the abundance of the *Metanosphaera* microbiota, which can utilise H_2_ to produce CH_4_ [52]. Due to the fact that only 2 mol H_2_ can generate 1 mol CH_4_, it may lead to a decrease in total gas production but an increase in methane production. In addition, an increase in the abundance of *WPS-2* and a decrease in the abundance of *Anaerotruncatus* were observed. *WPS-2* feeds on the intake of H_2_, CO, and CO_2_ [62,63,64], and the abundance of *Anaerotruncus* is positively correlated with natural gas production [65]. The comprehensive results of microbiology can explain why the total gas production in this study decreased with the increase in the addition of COS in the diet. Reducing gas production can to some extent alleviate greenhouse gas emissions. However, it should be noted that methane production may increase, and we need to increase the detection of gas components in subsequent experiments.

### 4.3. Effects of Adding Different Levels of COS to the Diet on Rumen Fermentation Parameters of Beef Cattle In Vitro

In this study, adding different levels of COS to the diet had different effects on the concentration and proportion of different VFA in the fermentation broth. Research has shown that the addition of chitosan can change the mode of rumen fermentation in ruminants, shifting the rumen from acetate fermentation to propionate fermentation, reducing the level of acetate in the rumen, increasing the level of propionate and reducing the A/P [50]. In addition, studies have shown that when the mode of rumen fermentation is changed, the total VFA concentration does not change [66,67], which is consistent with the results of our study. Meanwhile, in Liu’s previous study, it was mentioned that consistent with the findings in the present study, COS led to an increase in the content of butyrate, but there was no significant difference in the content and proportion of isobutyrate and valerate in their study. The microbiological results confirmed our findings. Among the communities with observed differences, endophytic microorganisms were able to ferment sugars into acetate and butyrate [68], leading to an increase in their abundance and an increase in acetate content. In previous studies, it was found that the main products of the *Subdoligranulum* community were small amounts of acetate and succinic acid [69], and a decrease in their relative abundance would lead to a decrease in acetate production. The relative abundance of *Anaerotruncus* is positively correlated with the concentrations of propionate, butyrate, and total volatile fatty acids [62]. Park [70] found that the microbial community of *Halomonas* mainly produces amylase and can promote the consumption of acetate. As its abundance increases, the consumption of acetate also increases. *Eubacteria* are associated with the production of VFA in the gastrointestinal tract, especially propionate and butyrate, which can convert monosaccharides into butyrate [71,72]. In our study, the increase in the relative abundance of *Endomicrobium*, *Eubacteria ruminantium_group*, and *Eubacteria Venturiosum_group* in the COS group accurately explained the increase in butyrate content, while the decrease in acetate content may be due to the decrease in the relative abundance of *Acetobacter* and *Subdoligranum*, as well as the increase in the abundance of *Halomonas*, which has a greater impact on acetate production than *Endomicrobium*. Under the joint influence of these microorganisms, the content of VFA components has changed. Meanwhile, the increased utilisation of CP, EE, and ADF by the rumen leads to the production of more VFA, and the increase in EE disappearance rate also produces more propionate [73,74,75], which explains the increase in propionate levels in this study. Overall, the main components of VFA are acetate, propionate, and butyrate, which account for more than 95% of the total VFA. Compared to these, changes in the concentration of other acids have little effect on the total VFA, with a decrease in acetate concentration and an increase in propionate. Under the combined influence of these factors, the reason why the total VFA concentration has not changed can be explained. Regarding NH_3_-N and MCP, the addition of COS to the diet significantly reduced the rumen NH_3_-N concentration (*p* < 0.01), and the 0.04% COS and 0.08% COS groups significantly increased the MCP content. The changes in NH_3_-N concentration are consistent with the results of the Goiri [50] and Zanferari [67] studies, but many studies on changes in MCP have produced inconsistent results. For example, the studies of Seankamthorn [76] and Rey [41] mentioned that the addition of chitosan has no significant effect on MCP. Whereas Gandra’s research also found that chitosan as an additive led to a decrease in MCP levels [77]. However, he also suggested that MCP synthesis may be positively correlated with rumen pH [77]. In this study, rumen pH increased significantly under the influence of COS (*p* < 0.05), which may have promoted the synthesis of MCP. In addition, the increase in rumen pH was also supported by the research of Kirwan [78], who pointed out that chitosan or COS contain NH_2_ groups, which can provide additional NH_4_^+^ after degradation in the rumen, thereby increasing rumen pH. COS leads to a decrease in the more acidic acetate and an increase in the less acidic propionate in VFA, which may also be the reason for the increase in pH. In summary, the transition from acetate fermentation to propionate fermentation in the rumen can increase the rumen pH, as confirmed by our results. Therefore, the addition of COS can maintain the rumen pH within a relatively safe range, reduce the risk of rumen acidosis, and lower concentrations of NH_3_-N can also promote the synthesis of rumen microorganisms, maintaining a relatively stable rumen environment.

### 4.4. Effects of Adding COS to the Diet on the Rumen Microbial Community of Beef Cattle In Vitro

In this study, at the phylum level, we observed *Bacteroidota*, *Proteobacteria,* and *Firmicutes* as communities with relatively high abundance. At the genus level, *Prevotella*, *Succinivibrionaceae UCG-002,* and *Rikenellaceae RC9_gut group* were communities with relatively high abundance, which is consistent with the dominant communities in rumen fluid in our previous study [30]. This indicates that our bovine rumen fluid fistula and RUSITEC system have good stability, and the data obtained can also be reproduced well in real animal experiments. At the phylum level, we also observed two distinct bacterial communities, *Latescriberota* and *WPS-2*, between the two treatment groups. *Latescoribacterota* has rarely been mentioned in previous studies, and its effects on animals have not yet been elucidated. However, Arcadi detected this community in the seawater and sedimentary rocks of Wurkano Island and pointed out that this community may be related to seawater acidification and has little correlation with the changes in various indicators in this study [79]. Several studies have shown that changes in diet structure or the addition of other substances to the diet can affect the relative abundance of *WPS-2* communities in animal bodies, which is beneficial to the animals. It has been suggested that the survival mode of *WPS-2* may be mainly through phagocytosis of H_2_, CO, and CO_2_, but the specific physiological function and effects of *WPS-2* on animals are still unclear [62,63,64]. At the genus level, we found a total of 17 different communities. Previous studies have mentioned that chitosan and its derivatives, due to the presence of R-NH_3_^+^ ions on the surface, can interact with negative ions on the microbial surface, causing peptidoglycan hydrolysis in the cell wall, leading to cell wall lysis, and have a stronger effect on Gram-positive bacteria than Gram-negative bacteria [80,81]. However, no significant difference was observed between Gram-negative and Gram-positive bacteria in this study. Studies have shown that the addition of chitosan can replace starch-degrading bacteria (*Bacteroidetes*) with fibre-degrading bacteria (*Firmicutes* and *Fibrobacteria*) in the rumen, resulting in a shift in rumen fermentation from the acetate mode to the propionate mode and a decrease in the relative abundance of methanogenic bacteria (*Proteobacteria*) [82]. We also observed similar results to Tong et al. such as an increase in the relative abundance of *Eubacterium_ruminantium_group*, *Eubacterium_ventriosum_group*, *Ruminococcus_gauvreauii_group* (*Firmicutes*) and the increase in relative abundance of *Acetobacter*, *Escherichia-Shigella* (*Proteobacteria*) and *Anaerotruncus* (*Bacteroidota*). The changes in rumen microbiota are closely related to the conditions of the rumen fluid donor animals, the diet and substrate composition, the equipment troubleshooting, and the in vitro method used in this experiment, and the rumen microbiota is also in a dynamic process in the rumen. We also found that the addition of COS led to an increase in the relative abundance of *Endomycobium*, which can reduce free energy wastage and optimise energy utilisation in the diet by generating ATP through substrate phosphorylation [77]. Increasing the relative abundance of *Ruminococcus* can improve rumen digestibility and regulate rumen function [53,54]. It also reduces the relative abundance of the conditionally pathogenic bacteria *Dorea* and *Escherichia Shigella*, which is important for maintaining animal health [83,84]. In summary, in this study, due to the chemical properties of COS, its addition led to changes in some microbial communities, resulting in changes in the disappearance rate of some nutrients and rumen fermentation indicators. At the same time, it regulated rumen function, reduced the abundance of harmful bacteria, and ensured rumen health.

## 5. Conclusions

In conclusion, different addition ratios of COS increased the disappearance rate of DM, CP, EE, and ADF in beef cattle to varying degrees. In terms of rumen fermentation, COS has been observed to increase the pH, MCP, and the content of propionate and butyrate. Additionally, the gas production, NH_3_-N, A/P, the content of acetate, isobutyrate, valerate, and protozoa count were significantly decreased, which gradually transitions rumen fermentation from an acetate mode to a propionate mode. The COS did not alter the diversity of rumen microbiota. However, it can cause changes in the relative abundance of microbial communities, including *Metanosphaera*, *Ruminococcus*, *Endomycobium*, and *Eubacterium*. Although gas production has decreased, there is a risk of an increase in CH_4_ content. Moreover, after combining the feed intake of beef cattle and the amount added in this study, the cost of COS used in the experiment was calculated to be about RMB 1.5/cattle/day. However, in the actual production process, with the continuous optimisation of extraction technology and large-scale purchases, the cost will only decrease. COS has good functions in improving nutrient digestion and maintaining rumen health. Therefore, we can consider COS as an additive that can be accepted by farmers. As the specific gas composition was not determined in this study, further work and animal experiments are needed in the future to objectively and comprehensively evaluate whether COS can be used as a safe and efficient additive.

## Figures and Tables

**Table 1:** Composition and nutrient levels of basal diets and fermentation substrates (DM basic).

Items	Content (%)
Ingredients	
Wheat straw	50
Corn	12.5
Soybean meal	4.35
Unhusked rice	14
Wheat bran	7
Oil bran	3.5
Sprayed corn husk	1
Soybean germ powder	1.5
Brow rice	1.5
Soybean husk	1
Rumen undegradable fat powder	0.15
Expanded urea	0.4
Unified bran	0.625
Premix ^a^	2.475
Total	100
Nutrient levels ^b^	
DM	91.89
CP	10.99
EE	3.00
NDF	69.95
ADF	32.26
Ash	10.69
Ca	0.81
P	0.25

^a^ Every 1 kg of premix contained 250,000 IU of vitamin A, 50,000 IU of vitamin D3, 800 IU of vitamin E, 0.9 g of CuSO_4_, 12 g of FeSO_4_, 14 g of MnSO_4_, 10 g of ZnSO_4_, 0.03 g of Na_2_SeO_4_, 0.02 g of KI, 0.02 g of CoCl_2_, 55 g of MgSO_4_. ^b^ The nutritional levels of the diets in the table are all measured values. DM, dry matter; CP, crude protein; EE, ether extract; NDF, neutral detergent fibres; ADF, acid detergent fibres; Ca, calcium; P, phosphorus.

**Table 2:** Effects of dietary different levels of COS on the rumen protozoa count of beef cattle.

Items	Dietary COS Level, Feeding Basis				SEM	*p*-Value
	CON	0.02% COS	0.04% COS	0.08% COS		
Number of Rumen Protozoa on Days 1–8 (10^5^/mL)						
Day 1 ^A^	7.86	7.86	7.86	7.86	0	/
Day 2	2.82	2.84	2.85	2.73	0.072	0.374
Day 3	1.13	1.22	1.21	1.15	0.057	0.357
Day 4	0.74	0.73	0.74	0.72	0.023	0.783
Day 5	0.67	0.68	0.68	0.64	0.029	0.441
Day 6	0.57 ^a^	0.58 ^a^	0.54 ^ab^	0.50 ^b^	0.024	0.015
Day 7	0.49 ^ab^	0.51 ^a^	0.46 ^ab^	0.44 ^b^	0.028	0.065
Day 8	0.47 ^a^	0.46 ^ab^	0.44 ^ab^	0.42 ^b^	0.020	0.144

In the table, the superscript “a–b” represents the differences in data between the treatment groups. Different letters indicate differences between the groups (*p* < 0.05), while the same or no letters indicate no significant differences (*p* > 0.05); SEM, standard error of the mean. ^A^ On the first day, all fermentation tanks were filled with a solution of fermentation broth and RUSITEC system buffer for rumen protozoa counting. This solution was evenly distributed throughout the tanks, ensuring that the protozoa count on the first day was consistent.

**Table 3:** Effects of adding different levels of COS to the diet on nutrient disappearance rates and total gas production of rumen nutrients in beef cattle.

Items	Dietary COS Level, Feeding Basis				SEM	*p*-Value		
	CON	0.02% COS	0.04% COS	0.08% COS		Main Effect	Linear	Quadratic
Nutritional Components Apparent Disappearance Rates (%)								
DM	77.44 ^c^	77.48 ^c^	77.81 ^b^	78.42 ^a^	0.151	<0.01	0.587	<0.01
OM	65.28 ^c^	65.32 ^bc^	65.66 ^b^	66.23 ^a^	0.165	<0.01	0.444	<0.01
CP	73.04 ^c^	73.67 ^bc^	73.99 ^b^	74.93 ^a^	0.305	<0.01	0.549	<0.01
EE	64.25 ^c^	64.63 ^c^	65.66 ^b^	66.23 ^a^	0.217	<0.01	0.749	<0.01
NDF	53.71	53.66	53.45	53.81	0.434	0.863	0.709	0.920
ADF	52.02 ^b^	52.27 ^b^	52.55 ^ab^	53.08 ^a^	0.361	<0.05	0.877	0.062
Total gas production (L/d)								
Day6	7.82 ^a^	7.74 ^a^	7.61 ^b^	7.52 ^b^	0.066	<0.01	<0.01	<0.05
Day7	7.86 ^a^	7.79 ^a^	7.64 ^b^	7.55 ^b^	0.068	<0.01	<0.01	<0.05
Day8	7.76 ^a^	7.69 ^ab^	7.55 ^bc^	7.47 ^c^	0.071	<0.01	<0.01	<0.05

In the table, the superscript “a–c” represents the differences in data between the treatment groups. Different letters indicate differences between the groups (*p* < 0.05), while the same or no letters indicate no significant differences (*p* > 0.05); SEM, standard error of the mean. DM, dry matter; OM, organic matter; CP, crude protein; EE, ether extract; NDF, neutral detergent fibre; ADF, acid detergent fibre.

**Table 4:** Effects of different levels of COS on rumen fermentation parameters of beef cattle.

Items	Dietary COS Level, Feeding Basis				SEM	*p*-Value		
	CON	0.02% COS	0.04% COS	0.08% COS		Main Effect	Linear	Quadratic
pH	6.42 ^c^	6.43 ^c^	6.46 ^b^	6.49 ^a^	0.007	<0.01	0.858	<0.01
NH_3_-N (mg/dL)	7.98 ^a^	7.93 ^a^	7.69 ^b^	7.35 ^c^	0.090	<0.01	0.739	<0.01
MCP (mg/mL)	0.59 ^b^	0.59 ^b^	0.61 ^ab^	0.62 ^a^	0.010	0.104	0.733	<0.01
Total VFA (mmol/mL)	112.53	113.09	113.83	113.43	1.249	0.104	0.591	0.557
Acetate (mmol/mL)	68.95 ^a^	68.15 ^ab^	67.37 ^ab^	66.70 ^b^	0.897	<0.01	0.584	0.07
Propionate (mmol/mL)	25.83 ^b^	27.13 ^a^	27.66 ^a^	27.66 ^a^	0.524	<0.01	0.059	<0.01
Isobutyrate (mmol/mL)	0.72 ^a^	0.69 ^b^	0.66 ^c^	0.65 ^c^	0.008	<0.01	0.272	<0.01
Butyrate (mmol/mL)	13.74 ^b^	13.76 ^b^	14.91 ^a^	15.23 ^a^	0.287	0.635	0.880	<0.01
Isovalerate (mmol/mL)	1.48	1.51	1.47	1.48	0.036	<0.01	0.410	0.467
Valerate (mmol/mL)	1.82 ^a^	1.85 ^a^	1.76 ^b^	1.71 ^b^	0.027	0.76	0.334	<0.01
Acetate/propionate	2.67 ^a^	2.51 ^b^	2.43 ^b^	2.42 ^b^	0.044	<0.01	0.042	<0.01

In the table, the superscript “a–c” represents the differences in data between the treatment groups. Different letters indicate differences between the groups (*p* < 0.05), while the same or no letters indicate no significant differences (*p* > 0.05); SEM, standard error of the mean. pH, potential of hydrogen; MCP, microbial protein.

## Data Availability

The data that support the findings of this study are available on request from the authors.
